# HDAC11 promotes renal fibrosis by induing partial epithelial-mesenchymal transition and G2/M phase arrest in renal epithelial cells

**DOI:** 10.1186/s10020-025-01367-3

**Published:** 2025-11-22

**Authors:** Yingjie Guan, Fengchen Shen, Liyuan Yao, Meiying Chang, Ting C. Zhao, Shougang Zhuang

**Affiliations:** 1 Department of Medicine, Rhode Island Hospital and Alpert Medical School, Brown University, Providence, RI R02903 USA; 2 Department of Nephrology, Shanghai East Hospital, Tongji University School of Medicine, Shanghai, 200120 China; 3 Departments of Surgery and Plastic Surgery, Rhode Island Hospital, Warren Alpert Medical School of Brown University, Providence, RI 02903 USA

**Keywords:** Histone deacetylase 11, Kidney fibrosis, Unilateral ureteral obstruction, Partial epithelial-mesenchymal transition, Smad3, Signal transducer and activator of transcription 3 Nuclear factor kappa B, FT895

## Abstract

**Background:**

Histone deacetylase 11 (HDAC11) is the sole member of class IV HDACs, implicated in tumor growth, immune regulation, and oxidative stress injury. Its specific role in renal fibrosis and underlying mechanisms remains unclear.

**Methods:**

The global knockout of HDAC11 mice and FT895, a selective inhibitor of HDAC11, were utilized to assess the role of HDAC11 in renal fibrosis following unilateral ureteral obstruction (UUO) injury in mice. Immunostaining was employed to analyze renal expression of HDAC11 and infiltration of macrophages. Immunoblot analysis was used to analyze the expression and/or phosphorylation of proteins associated with partial epithelial-mesenchymal transition (pEMT) in the kidney and cultured renal proximal tubular cells (RPTCs). RT-PCR was used to analyze the expression of various proinflammatory cytokines.

**Results:**

HDAC11 was predominantly expressed in renal epithelial cells, with its expression increasing in the kidney following UUO. This upregulation correlated with excessive collagen deposition and was associated with increased levels of fibronectin, collagen I, and α-smooth muscle actin, alongside reduced E-cadherin expression. Both global deletion of HDAC11 and treatment with the selective inhibitor FT895 significantly reduced collagen accumulation and the expression of fibronectin and collagen I, while preserving E-cadherin levels. HDAC11 inhibition also led to a decrease in histone H3 phosphorylation at serine 10, a marker of G2/M cell cycle arrest, and reduced the expression of Snail and Twist—key transcription factors involved in pEMT. Similar effects were observed in TGF β1-stimulated RPTCs in vitro treated with FT895 or subjected to HDAC11 silencing via siRNA. Additionally, FT895 treatment attenuated the expression of multiple pro-inflammatory cytokines and reduced macrophage infiltration in obstructed kidneys. Both pharmacological inhibition and genetic ablation of HDAC11 suppressed activation of profibrotic signaling pathways, including Smad3, STAT3, and NF-κB, in both in vitro and in vivo models.

**Conclusions:**

These findings indicate that HDAC11 is crucial for renal fibrosis development by promoting pEMT and G2/M phase cell cycle arrest in renal epithelial cells through multiple profibrotic signaling pathways. Therefore, targeting HDAC11 may be a promising therapeutic strategy to alleviate renal fibrosis.

**Supplementary Information:**

The online version contains supplementary material available at 10.1186/s10020-025-01367-3.

## Background

Chronic kidney disease (CKD) is a global public health problem. It affects more than 20 million people in the United States alone (Lerner et al. 2017). Regardless of the etiologies, CKD would progress to end stage of renal disease (Bello et al. 2024; Lerner et al. 2017). In the past several decades, studies have revealed that the common pathway underlying CKD is kidney fibrosis, which is characterized by renal interstitial fibroblast activation, extracellular matrix (ECM) accumulation, inflammatory responses and destruction of normal structure (Bello et al. 2024; Reiss et al. 2024). Currently, there is still no specific treatment for targeting fibrosis. This enormous unmet medical need calls for better understanding of the mechanism underlying fibrotic CKD.

Increasing evidence has revealed that partial epithelial-mesenchymal transition (pEMT) is a critical pathological process that initiates activation of renal interstitial fibroblasts and production of ECM components in the kidney following various injuries. (Grande et al. 2015; Lovisa et al. 2016; Sheng and Zhuang 2020) pEMT is defined as the state in which epithelial cells express markers of both epithelial and mesenchymal cells, but remain associated with the basement membrane (Lovisa et al. 2016; Sheng and Zhuang 2020). The renal epithelial cells with pEMT are arrested at the G_2_/M phase of cell cycle, and able to generate a large amount of profibrotic growth factors/proinflammatory factors. Subsequently, these factors are released into the renal interstitium where induces the transformation of fibroblasts to active fibroblasts (myofibroblasts) (Lovisa et al. 2016; Sheng and Zhuang 2020). Several signaling pathways such as transforming growth factor-b/Smad3 (TGFb/Smad3), signal transducer and activator of transcription 3 (STAT3) and some transcription factors such as Snail and Twist, have been identified to participate in the process of pEMT in the injured kidney (Lovisa et al. 2016; Pang et al. 2010; Sheng and Zhuang 2020). Recently, a number of post-translational modifications, including protein acetylation, have also been recognized to contribute to this process.

Protein acetylation is one of the post-translational modifications that occurs in both histone and non-histone proteins (Shen and Zhuang 2022). Histone acetylation often occurs at positively charged lysine residues which weakens the DNA-histone interactions, thus opening the chromatin and facilitating transcription (Shen and Zhuang 2022). For example, acetylation of lysine 9 on histone 3 (H3K9ac) correlates with transcription activation (Nozaki and Kanai 2021). Acetylation also involves in regulation of many non-histone proteins, including transcription factors, transcriptional coactivators and nuclear receptors (Shvedunova and Akhtar 2022). Acetylation is positively regulated by histone acetyltransferases and negatively by histone deacetylates (Shen and Zhuang 2022; Wang et al. 2022). As such, either activation of histone acetyltransferases or inactivation of histone deacetylates is able to promote protein acetylation (Shen and Zhuang 2022). To date, numerous histone acetyltransferases and histone deacetylates have been identified. Depending on their sequence homology, HDACs are divided into four categories with 11 isoforms: class I HDACs (HDAC1-3,8), class II HDACs (HDAC 4–7, 9; 10), class III [sirtuin (SIRT)1–SIRT7], and class IV (HDAC11). The various HDACs exhibit distinct functions, unique cellular and organ distribution, as well as discrete physiological and pathological effects (Shen and Zhuang 2022; Tang and Zhuang 2019).

Previous studies have shown that HDACs play a key role in the development of various kidney diseases (Shen and Zhuang 2022). Certain HDAC isoforms, such as HDAC3, 4, 6, 8 and 9, are upregulated in injured kidneys and contribute to renal fibrosis (Chen et al. 2020; Pang et al. 2009; Shen and Zhuang 2022; Zhang et al. 2020). These findings were obtained using either class- or isoform-specific inhibitors or conditional deletion techniques (Shen and Zhuang 2022). A recent study showed that inhibition of HDAC11 by quisinostat attenuated renal fibrosis induced by unilateral ureteral obstruction (UUO) (Mao et al. 2020). However, quisinostat is not a specific HDAC11 inhibitor and can also target several other HDACs with equivalent potency—all of which have been shown to play regulatory roles in renal fibrosis, (Arts et al. 2009) thus, it remains uncertain about the role and mechanisms of HDAC11 in renal fibrosis.

In this study, we employed globally deleted HDAC11 mice and FT895, a highly selective inhibitor of HDAC11, (Martin et al. 2018) to investigate the role and mechanism of HDAC11 in renal fibrosis. Our results demonstrate that either global deletion of HDAC11 or administration of FT895, attenuated renal fibrosis, pEMT and G2/M phase arrest in renal epithelial cells in a murine model of UUO. Both FT895 treatment and siRNA-mediated silencing of HDAC11 also inhibited TGF-β1 induced pEMT in cultured murine renal proximal tubular epithelial cells (RPTCs). Based on the data, we concluded that HDAC11 is critically involved in renal fibrogenesis through a mechanism involving the suppression of EMT and G2/M phase arrest in renal epithelial cells.

## Methods

### Chemicals and antibodies

Antibodies for collagen I, α-smooth muscle actin (α-SMA), and Twist were sourced from Abcam (Cambridge, MA, USA). Antibodies for glyceraldehyde 3-phosphate dehydrogenase (GAPDH), α-Tubulin, and HDAC11 were purchased from Santa Cruz Biotechnology (Dallas, TX, USA). The antibody for Snail was from Biorbyt (Durham, NC, USA), and the antibody for histone H3 was obtained from Sigma-Aldrich (St. Louis, MO, USA). Antibodies for fibronectin and phospho-histone H3 were from Novus Biologicals (Centennial, CO, USA). siRNA specific for HDAC11 was purchased from Invitrogen (MA, USA). FT895 was acquired from Cayman Chemical (MA, USA). The anti–F4/80 antibody was purchased from Abcam (Waltham, MA, USA). All other items, including antibodies against phospho-STAT3, STAT3, phospho-NF-κB (p65), NF-κB (p65), Snail1, acetyl-histone H3, phospho-histone H3 (Ser10; for immunofluorescence staining), and E-cadherin, were obtained from Cell Signaling Technology (Danvers, MA, USA).

### Animals and experimental design

Global HDAC11 knockout mice (HDAC11^−/−^) were obtained from Shanghai Model Organism. These mice were found to be viable and developed normally, as reported in previous studies (Nunez-Alvarez et al. 2021). The UUO model was established in male C57B6 black mice weighing 20-25 g (The Jackson Laboratory, Bar Harbor, ME, USA) in accordance with our previous studies (Pang et al. 2009). In brief, a flank incision was made to expose the abdominal cavity, and the left ureter was isolated and ligated. The contralateral kidney served as a control. To investigate the role of HDAC11 in renal fibrosis, FT895 (5 mg/kg) was administered by intraperitoneal injection in 0.5 mL of a 10% DMSO–FBS mixture immediately after ureteral Ligation and then once daily for 6 consecutive days. The dosage of HDAC11 was chosen based on a previous report by Rau et al. (Martin et al. 2018). For the UUO-alone group, mice were injected with an equivalent amount of 10% DMSO–FBS mixture. At 7 days post-ligation, mice were euthanized and both obstructed and contralateral non-obstructed kidneys were harvested for various analysis. All animal study procedures were approved by the Lifespan Institutional Animal Care and Use Committee and conducted in compliance with the NIH Guide for the Care and Use of Laboratory Animals. The animals had ad libitum access to standard rodent chow and water.

### Western blot analysis

Cell and mouse kidneys were homogenized in RIPA lysis buffer (150 mM NaCl, 50 mM Tris–HCl pH 8, 1% NP-40, 0.5% sodium deoxycholate, 0.1% SDS) supplemented with a protease inhibitor cocktail (Complete™, Roche). The total protein amount was determined using the BCA assay (Thermo Scientific), and equal amounts of lysates for each sample were separated by SDS-PAGE and transferred to nitrocellulose membranes. Following a 1-h incubation with 5% nonfat milk at room temperature, the membranes were then exposed to a primary antibody overnight at 4 °C followed by an appropriate horseradish peroxidase-conjugated secondary antibody for 1 h. Bound antibodies were visualized using chemiluminescence detection. The catalog number and dilution ratio for each primary and secondary antibody are listed in Supplemental Table 1. The semi-quantitative analysis of different proteins was conducted using ImageJ software (NIH). Densitometry analyses were quantified based on the intensity (density) of the band, which was calculated by the area and pixel value of the band. The quantification data are presented as a ratio between the target protein and loading control (housekeeping protein).

### Histology and Immunohistochemistry analysis

Mouse kidney samples were fixed in 10% formalin (Sigma-Aldrich) overnight and embedded in paraffin. 5-μm thick sections were used for immunohistochemistry. Masson trichrome staining was carried out according to the procedure described in previous studies (Pang et al. 2010). To quantitatively assess renal fibrosis, the collagen tissue area was measured using Image Pro-Plus Software (Media Cybernetics, Rockville, MD, USA) by drawing a Line around the perimeter of the positive staining area, and calculating and graphing the mean ratio to each microscopic field. For immunofluorescence staining, sections were deparaffinized, rehydrated and antigen retrieved at 98 °C for 10 min in 10 mM citrate buffer pH 6. The tissue sections were incubated with 5% normal goat serum, 5% BSA in TBST for 30 min prior to overnight incubation with the primary antibody. The primary antibodies used and their dilution rates are listed in Supplemental Table 2. Eight to ten randomly selected fields at a magnification of 400 × on each section were then digitally photographed and scored in a blinded fashion via computerized morphometric analysis.

### Quantitative real–time PCR analysis

RNA extraction and quantitative real-time polymerase chain reaction (PCR) were conducted following the procedure outlined in our previous studies (Wang et al. 2018). Kidneys were homogenized, and total RNA was extracted according to the manufacturer's instructions (Qiagen). cDNA synthesis was carried out using the Bio-Rad cDNA synthesis kit as per the manufacturer's protocol. The mRNA expression levels of the listed genes were quantified by real-time PCR using the SYBR Green PCR Master mix (Qiagen) and normalized to the expression of GAPDH housekeeping gene. Relative transcription levels were calculated as previously described (Pang et al. 2010). The genes and primer sequences are identical to those previously utilized (Pang et al. 2010).

### Cell culture and treatment

Mouse renal proximal tubular epithelial cells (RPTCs) were cultured in DMEM medium supplemented with 10% heat–inactivated fetal bovine serum and penicillin/streptomycin (100 ~ g/ml) at 37 °C and 5% CO2. To assess the impact of FT895, various doses of FT895 were directly added to the sub-confluent RPTCs, followed by a 24-h incubation period. For the induction of EMT, RPTCs were starved for 24 h in serum-free medium and then exposed to 5 ng/ml TGFβ1 (R&D Systems) for 48 h in the presence or absence of FT895. The control for TGFβ1 treatment utilized a vehicle consisting of 4 mM HCl in H2O with 1 mg/ml BSA. To transfect siRNA into RPTC cells, we seeded cells to a confluence of 60–70% in antibiotic-free medium and transfected them with siRNA specific to HDAC11 (100 pmol) using Lipofectamine 2000 (Thermo Fisher Scientific, Waltham, MA, USA), following the manufacturer's instructions. In parallel, scrambled siRNA (100 pmol) was used as a control for off-target changes in RPTC cells. Twenty-four hours after transfection, the medium was changed and cells were incubated in the absence or presence of TGFb1 for an additional 48 h before being harvested for analysis.

### Statistical analyses

The data are presented as means ± standard deviation (SD). Statistical analyses of immunohistochemical/immunofluorescence quantifications and qPCR analysis were performed using one-way ANOVA or unpaired or two-tailed Student’s t-test with Welch’s correction in GraphPad Prism software (GraphPad Software). Statistical significance was defined as *P* < 0.05.

## Results

### Pharmacological inhibition of HDAC11 by FT895 attenuates renal fibrosis in a murine model of UUO

**Fig. 1:**
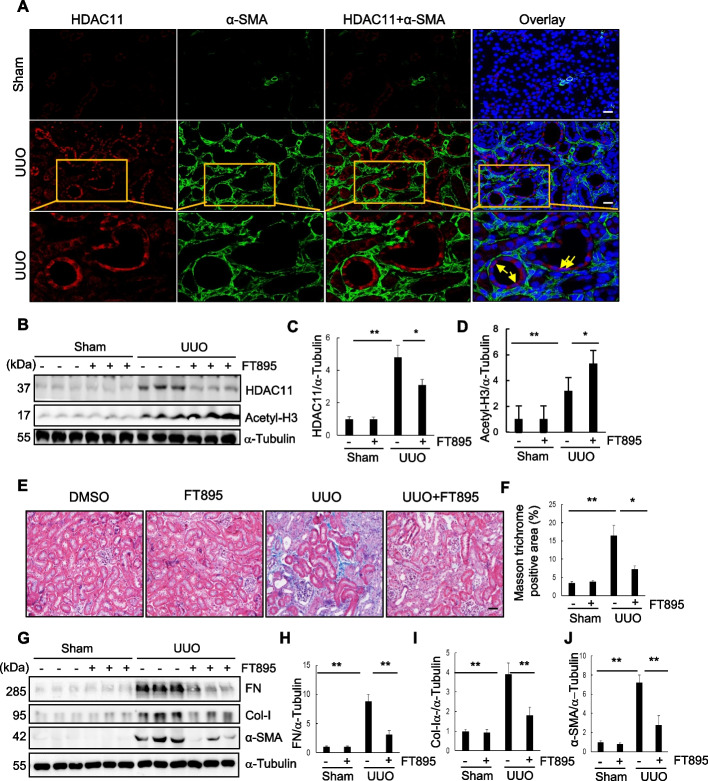
Expression of HDAC11 and the effect of FT895 on renal fibrosis in obstructed kidneys. **A** Photomicrographs illustrate co-staining of a-SMA and HDAC11 in the tissue section of the obstructed kidney (original magnification, 400 X). Arrows indicate regions of nuclear localization of HDAC11. **B** The prepared tissue lysates from sham-operated or obstructed kidneys of mice administered with or without FT895 were subjected to immunoblot analysis with antibodies against HDAC11, acetylated histone 3, or α-Tubulin. **C** and **D** The levels of HDAC11, acetylated histone 3, or α-Tubulin were quantified by densitometry, and HDAC11 (**C**) and acetylated histone 3 (**D**) levels were normalized to a-Tubulin. Values are means ± SD (*n* = 6). **P < 0.01, *P < 0.05. **E** Photomicrographs illustrating Masson trichrome staining (blue) of kidney tissue (original magnification, 400 x). **F** The Masson trichrome–positive tubulointerstitial area was analyzed relative to the whole area from 10 random cortical fields. Data are represented as means ± SD (*n* = 6). **G** Kidney tissue lysates were subjected to immunoblot analysis with antibodies against fibronectin (FN), collagen I (Col), α-SMA, or α-Tubulin. **H**–**I** Expression levels of FN, Col-I, α-SMA, or α-Tubulin were quantified by densitometry, and the levels of FN (**H**), Col-I (**I**), and a-SMA (**I**) were normalized with a-Tubulin. Values are means ± SD (*n* = 6). *P < 0.05, **P < 0.01. Scale bars = 50 µm

To investigate the role of HDAC11 in the development of renal fibrosis, we first examined the renal expression of HDAC11 in a Murine model of renal fibrosis induced by UUO. Following UUO surgery, kidneys were collected at day 7 and co-stained with anti–HDAC11 and α-SMA bodies to visualize HDAC11. As depicted in Fig. 1A, minimal expression of HDAC11 was observed in sham-operated kidneys, whereas abundant expression was evident in kidneys with UUO injury. Co-staining of HDAC11 with α-SMA indicated that HDAC11 was present in renal tubular cells but not in α-SMA–positive interstitial fibroblasts. Further, co-staining with DAPI, a nuclear dye, revealed that HDAC11 was primarily localized in the cytoplasm; however, a small amount was also detectable within the nucleus, particularly at the nuclear periphery, which appeared purple due to the overlap of HDAC11 and DAPI signals. Additionally, co-staining of HDAC11 with acetyl-histone H3 showed widespread expression of acetyl-histone H3 in renal tubular cells of both sham-operated and UUO-operated kidneys (Supplemental Fig. 1 A). Similarly, immunoblot analysis results demonstrated slight detection of HDAC11 in sham‐operated kidneys but a dramatic increase of its expression at 7 days after UUO injury (Fig. 1B,C).

**Fig. 2:**
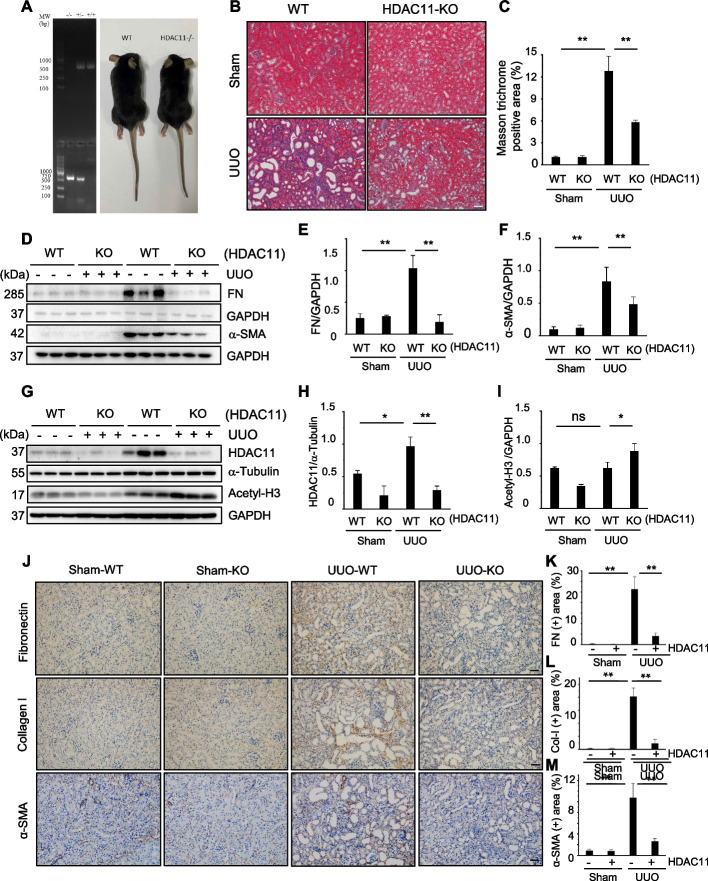
Global deletion of HDAC11 attenuates renal fibrosis in mice. A PCR analysis of HDAC11 expression in RNA extracted from HDAC11 WT and KO mouse tissue (Left). The DNA molecular weight size marker is shown in the far-left lane; similar size of HDAC11 WT and KO mice (right). **B** Photomicrographs illustrating Masson trichrome staining (blue) of kidney tissue (original magnification, 400 x). **C** The Masson trichrome–positive tubulointerstitial area was analyzed relative to the whole area from 10 random cortical fields. **D**, **G** Kidney tissue lysates were subjected to immunoblot analysis with antibodies against proteins as indicated. All these proteins were quantified by densitometry, and Fibronectin (FN) (**E**), α-SMA (**F**), HDAC11 was normalized with α-Tubulin (**H**), and acetyl-histone H3 (Acetyl-H3) was normalized GAPDH (**I**), respectively. **J** Photomicrographs showing immunohistochemical staining for fibronectin, collagen I, and α-SMA (original magnification, 200 x). The positive staining areas for fibronectin (**K**), collagen-I (**L**), and α-SMA (**M**) were quantified as a percentage of the total area from 10 random cortical fields. Data are represented as means ± SD (*n* = 6). *P < 0.05, **P < 0.01. Scale bars = 50 µm

To investigate the role of HDAC11 in renal fibrosis, FT895, a highly selective inhibitor of HDAC11, was employed. This compound demonstrates over 1000-fold selectivity compared to other HDACs (Martin et al. 2018). As illustrated in Fig. 1 E, extensive deposition of collagen fibrils within the interstitial space was evident by an increase in positive areas on Masson trichrome staining in the kidney following UUO injury. Semiquantitative analysis revealed over a fourfold increase in ECM component deposition within obstructed kidneys compared to sham controls; administration of FT895 significantly reduced ECM deposition (Fig. 1F). Immunoblot analysis of whole kidney lysates further demonstrated increased expression levels of α‐SMA, collagen I, and fibronectin—three key fibrotic markers—in the injured kidneys. Administration of FT895 significantly reduced their expression (Figs. 2G–J). Consistent with these findings, similar results were observed by immunohistochemical staining using individual antibodies against these markers (Supplemental Fig. 1 B-E). The effectiveness of FT895 was evident by reduced expression of HDAC11 and reciprocally increased acetylation of histone H3 (Fig. 1B-D). These findings suggest that pharmacological inhibition of HDAC11 effectively mitigates the progression of renal fibrosis.

### Global deletion of HDAC11 attenuates renal fibrosis following UUO injury

To validate the functional role of HDAC11 in renal fibrosis, we further investigated the impact of loss of HDAC11 on renal fibrosis by using mice with global deletion of HDAC11 (HDAC11^−/−^or KO mice). The correct genotyping of wild-type (WT) and KO mice was assessed by PCR (Fig. 2A). Along with previous report, the loss of HDAC11 mRNA in KO mice by RT-PCR. HDAC11^−/−^ mice were viable and developed normally (Nunez-Alvarez et al. 2021), exhibiting similar size and appearance to WT type mice (Fig. 2A) Immunoblot analysis demonstrated increased expression of HDAC11 in the kidney of WT mice and reduced expression of it in the kidney of HDAC11^−/−^ mice following UUO injury, while expression of acetyl histone H3 was increased in the kidney of HDAC11^−/−^ mice (Fig. 2G- I). Immunofluorescent staining demonstrated increased expression of HDAC11, primarily in renal tubular cells of WT mice following UUO injury, while global deletion of HDAC11 markedly reduced its expression (Supplemental Fig. 2 A). Masson’s staining showed a lower deposition of collagen fibrils in the injury kidney of HDAC11^−/−^ mice relative to that in WT mice. Semiquantitative analysis also revealed reduced positive areas of Masson trichrome staining in the injured kidney of HDAC11^−/−^ mice compared with WT mice (Fig. 2B, C). Immunoblot analysis of fibrotic markers indicated that depletion of HDAC11 reduced the expression of fibronectin to the basal levels, and largely reduced the expression of α-SMA in UUO injured kidneys (Fig. 2D-F). Immunohistochemical staining also demonstrated that global deletion of HDAC11 reduced the expression of fibronectin, α-SMA, and collagen I in the kidney following UUO injury (Fig. 2J-M). These results confirmed that HDAC11 is an indispensable driver for renal fibrogenesis following UUO injury.

### Inhibition of HDAC11 with FT895 or deficiency of HDAC11 represses pEMT and arrest of epithelial cells at G2/M phase following UUO injury

**Fig. 3:**
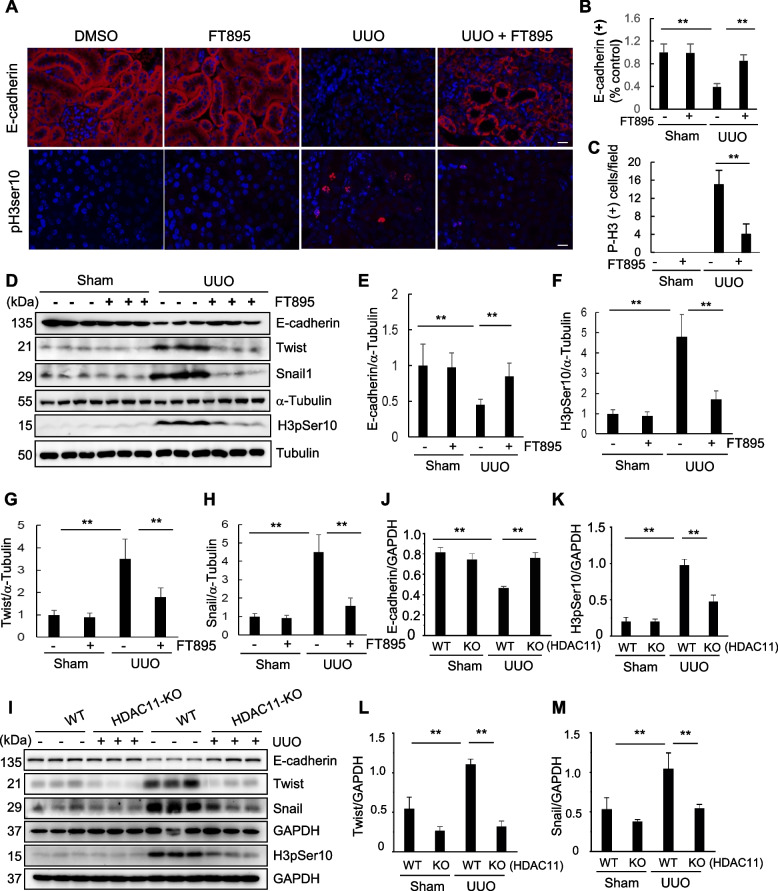
Pharmacological or genetic blockade of HDAC11 inhibits partial EMT and renal epithelial cells arrested in the G2/M phase of the cell cycle in obstructed kidneys. **A** Photomicrographs illustrate staining of E-cadherin and pH3Ser10 in the tissue section of the kidney after treatments as indicated (original magnification, 400x). The tubular cells with positive staining of E-cadherin (**B**) and pH3Ser10 (**C**) were calculated in 20 high-power fields and expressed as means ± SD. **P < 0.01. Kidney tissue lysates were subjected to immunoblot analysis with antibodies against proteins as indicated (**D**, **I**). Expression levels of E-cadherin (**E**, **J**), pH3Ser10 (**F**, **K**), Twist (**G**, **L**), Snail (**H**, **M**) were quantified by densitometry and normalized with GAPDH or a-Tubulin as indicated. Values are means ± SD (*n* = 6). **P < 0.01. Scale bars = 50 µm

The sustained pEMT and subsequent arrest of renal tubular epithelial cells at the G2/M phase of the cell cycle are essential for the development of renal fibrosis (Lovisa et al. 2016; Sheng and Zhuang 2020). The loss of the adherens junction protein E-cadherin and expression of phosphorylated histone H3 at serine 10 (H3pSer10) are commonly used as markers to characterize the presence of EMT and G2/M arrest, respectively (Lovisa et al. 2016; Sheng and Zhuang 2020). As HDAC11 is predominantly expressed in renal tubular cells as shown in Fig. 1, we investigated its potential role in mediating pEMT and G2/M arrest. Immunostaining results clearly demonstrated abundant expression of E-cadherin in renal tubular cells from sham and FT895 alone treated kidneys, with a reduction observed in the kidney following UUO injury. However, FT895 treatment largely restored E-cadherin expression. In contrast, H3pSer10(+) cells were not observed in sham and FT895 alone treated kidneys; however, this population of cells was increased in injured kidneys but reduced by FT895 treatment (Fig. 3A-C). Similar results were obtained when the protein levels of E-cadherin and H3pSer10 were examined through Western blot analysis (Fig. 3D-F).

Twist and Snail are two major transcriptional factors that repress the expression of epithelial genes such as E-cadherin and activate mesenchymal genes, (Sheng and Zhuang 2020) we thus investigated the impact of FT895 on their expression in the kidney with UUO injury. As depicted in Fig. 3D, G-H, their expression was barely detected in sham and FT895 alone treated kidneys but increased in UUO injured kidneys. Administration of FT895 largely reduced their expression levels. Similarly, HDAC11 deficiency substantially preserved the expression of E-cadherin while inhibiting that of Twist and Snail as well as H3pSer10 in the injured kidneys (Fig. 3J-M).

Taken together, these data suggest that HDAC11 plays an essential role in promoting the pEMT and arrest of epithelial cells at G2/M phase of cell cycle following fibrotic injury initiated by UUO.

### Blocking HDAC11 by FT895 or siRNA silencing inhibits TGFβ1‐induced EMT in RPTCs

**Fig. 4:**
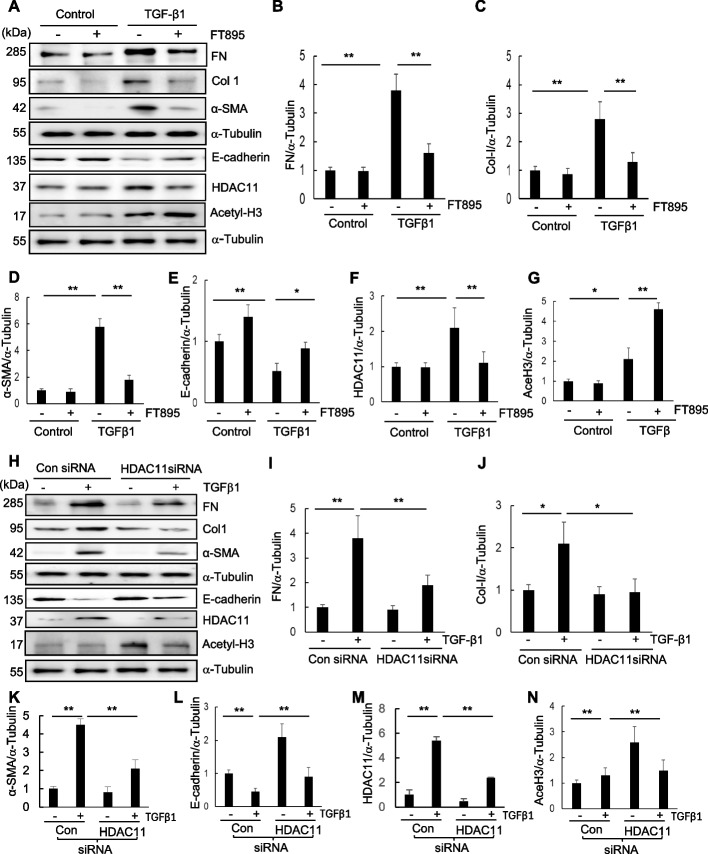
Treatment with FT895 or knockdown of HDAC11 with siRNA inhibits renal EMT. **A**-**G** Serum-starved RPTCs were pretreated with 10 µM FT895 for 1 h and then exposed to TGF-β1 (5 ng/ml) for an additional 24 h. (**H**-**N**) Serum-starved RPTC cells were transfected with siRNA targeting HDAC11 or control siRNA and then incubated in TGF-β1 (5 ng/ml) for an additional 24 h. Cell lysates were then prepared and subjected to immunoblot analysis with antibodies against proteins as indicated. Expression levels of fibronectin (FN) (**B**, **I**), collagen (Col-I) (**C**, **J**), a-SMA (**D**, **K**), E-cadherin (**E**, **L**), HDAC11 (**F**, **M**), or acetylated H3 (Ace-H3) (**G**, **N**) were quantified by densitometry and normalized with α-Tubulin, respectively. Values are the mean ± SD of at least 3 independent experiments. *P < 0.05, **P < 0.01

Previous studies have demonstrated that cultured renal epithelial cells exposed to TGF-β1 undergo the pEMT, characterized by downregulation of E-cadherin and upregulation of α-SMA, along with increased expression of fibronectin and collagen 1 (Zou et al. 2023), (Humphreys et al. 2010). To investigate the potential involvement of HDAC11 in regulating this process, we evaluated the impact of FT895 and HDAC11 siRNA on the expression of these proteins in cultured RPTCs in response to TGFβ1. Treatment with FT895 inhibited the TGFβ1-induced increases in α-SMA, collagen I, and fibronectin, while also preventing the TGFβ1-induced decrease in E-cadherin (Fig. 4A-E). This effect occurred at a dose that reduced HDAC11 expression to basal levels and reciprocally increased acetyl-histone H3 expression (Fig. 4A, F-G). Similar results were also demonstrated in RPTCs through siRNA-mediated silencing of HDAC11 (Fig. 4H-L). A successful knockdown of HDAC11 was observed in cells transfected with HDAC11 siRNA compared to those transfected with control siRNA, which was accompanied by a slight increase in acetyl-histone H3 expression (Fig. 4H, M–N). Collectively, these findings support a conclusion that HDAC11 is involved in the development of the EMT in renal epithelial cells.

### HDAC11 is involved in the phosphorylation of Smad3 and STAT3 and expression of Smad7, Klotho and BMP-7 in UUO‐injured kidney and cultured renal tubular cells

**Fig. 5:**
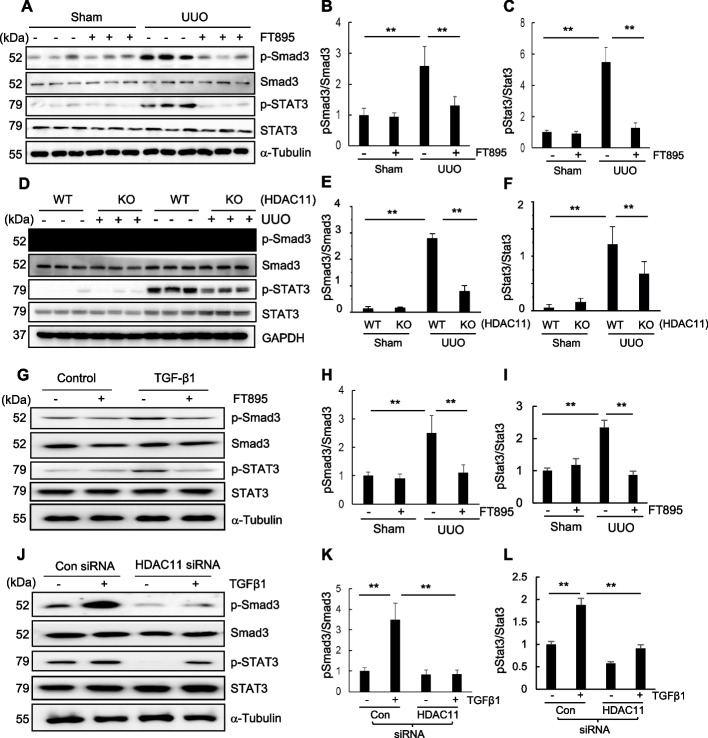
Pharmacological and genetic inhibition of HDAC11 reduces phosphorylation of Smad and STAT3 in obstructed kidneys and cultured renal epithelial cells. Kidney tissue lysates were prepared and subjected to immunoblot analysis with antibodies to proteins as indicated (**A**-**F**). Serum-starved RPTCs were treated with 10 µM FT895 for 1 h followed by exposure of cells to TGFβ1 (5 ng/ml) for an additional 24 h (**G**, **F**) or transfected with siRNA targeting HDAC11 or control siRNA and then incubated in TGFβ1 (5 ng/ml) for an additional 24 h (**J**-**L**). Cell lysates were subjected to immunoblot analysis with antibodies against proteins as indicated (**G**, **L**). Expression levels of the proteins were quantified by densitometry. Phospho-Smad3 was normalized to its total Smad3 protein level (**B**, **E**, **H**, **K**); Phospho-STAT3 was normalized to its total STAT3 protein level (**C**, **F**, **I**, **L**). Values are means ± SD (*n* = 6) for immunoblot of kidney lysates; values are the mean ± SD of at least 3 independent experiments for immunoblot of cell lysates. **P < 0.01

**Fig. 6:**
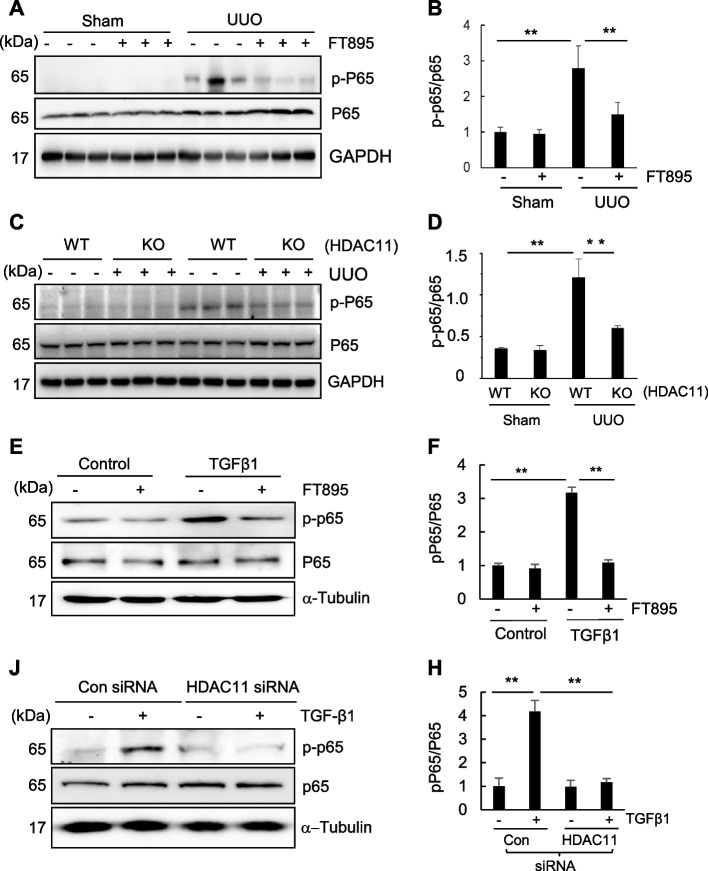
Inhibition or knockdown of HDAC11 inhibits activation of STAT3 and NF-κB signaling in obstructed kidneys and cultured renal epithelial cells. **A**, **C** Kidney tissue lysates were prepared from mice with various treatments as indicated and subjected to immunoblot analysis with antibodies against phospho–NF- κB (p-P65), or total P65. All of those proteins were quantified by densitometry, and phospho–NF-κB (**B**, **D**) were normalized to its total protein levels. Values are means ± SD (*n* = 6). **P < 0.01. Serum-starved RTPCs were treated with 10 μM FT895 for 1 h (**E**, **F**) or transfected with siRNA targeting HDAC11 or scrambled siRNA (control siRNA) for 12 h (**J**, **H**), followed by exposure of cells to TGFβ1 (5 ng/ml) for an additional 48 h. Cell lysates were subjected to immunoblot analysis with antibodies against p-P65, P65 or a-Tubulin (**E**, **J**). Expression levels of the proteins were quantified by densitometry, and p–P65 were normalized to its total protein levels (**F**, **H**). Values are the mean ± SD of at least 3 independent experiments. **P < 0.01

To elucidate the underlying mechanism by which HDAC11 induces EMT and renal fibrosis in the UUO model, we investigated the effect of HDAC11 inhibition on the phosphorylation of Smad3 (p-Smad3) and STAT3 (p-STAT3) in the kidney and cultured RTPCs. Smad3 is a key downstream factor of TGFβ1, and STAT3 acts downstream of many membrane receptors associated with renal fibrosis, including TGF βreceptors (Yuan et al. 2022). Immunoblot analysis showed increased expression levels of p-Smad3 and p-STAT3 in the kidney with UUO injury, which was abolished by administration of FT895. Total Smad3 and STAT3 expression levels remained unchanged in kidneys with or without UUO injury and were not affected by FT895 treatment (Fig. 5A–C). Similarly, UUO injury led to increased phosphorylation of Smad3 and STAT3 in WT mice, whereas HDAC11 deficiency significantly reduced their phosphorylation levels. In contrast, the total expression levels of Smad3 and STAT3 were not affected by HDAC11 deletion (Fig. 5D-F). In cultured RPTCs, TGFβ1 treatment also induced an increase in the phosphorylation of p-Smad3 and p-STAT3, and treatment with either FT895 or HDAC11 siRNA abolished their phosphorylation without altering total Smad 3 and STAT3 levels (Fig. 6G-L). Therefore, these data suggest that HDAC11 is required for activating TGFβ/Smad and STAT3 signaling during renal fibrogenesis and phenotypic transition of renal epithelial cells.

As our previous studies have shown that several anti-fibrotic molecules, such as Smad7, Klotho and BMP-7 are regulated by different HDAC isoforms in the kidney following chronic injury, (Chen et al. 2020; Lin et al. 2017; Xiong et al. 2019; Zhang et al. 2020) we further examined the effect of HDAC11 deficiency on the expression of these molecules. As shown in Supplemental Fig. 3, UUO injury induced downregulation of these molecules, whereas global deletion of HDAC11 largely restored their expression, suggesting that HDAC11-mediated repression of these two molecules may also contribute to renal fibrogenesis.

### FT895 inhibits increased phosphorylation of NF‐κB in the kidney after obstructed injury

Studies have demonstrated that activation of the nuclear factor kappa B (NF-κB) signaling pathway contributes to renal inflammation and fibrogenesis and HDAC11 mediates inflammatory response (White et al. 2020; Yanginlar and Logie 2018). We thus hypothesized that HDAC11 may play a role in the activation of this signaling pathway in the kidney upon injury. As depicted in Fig. 6A,B, basal levels of phosphorylated NF‐κB p65 (Ser536) (p-NF‐κB p65) was observed in the sham-operated kidney, with significantly increased levels detected in the kidney following obstructive injury. Treatment with FT895 reduced phosphorylation of NF‐κB in the injured kidney without significantly changing the expression levels of total NF‐κB compared to normal kidneys. The elevated expression levels of p-NF-κB p65 were also evident in the injured kidney of wild-type mice and decreased in the injured kidney of HDAC11 (+/+) mice (Fig. 6C, D). Additionally, inhibition of HDAC11 by FT895 or transfection of HDAC11-specific siRNA blocked TGFβ1-induced NF-κB phosphorylation in cultured RPTC cells (Fig. 6E–H). Therefore, HDAC11 is essential for the activation of NF-κB signaling pathways during renal fibrosis and renal epithelial cell transformation.

### Inhibition of HDAC11 attenuates and macrophage infiltration in the kidneys after UUO injury

**Fig. 7:**
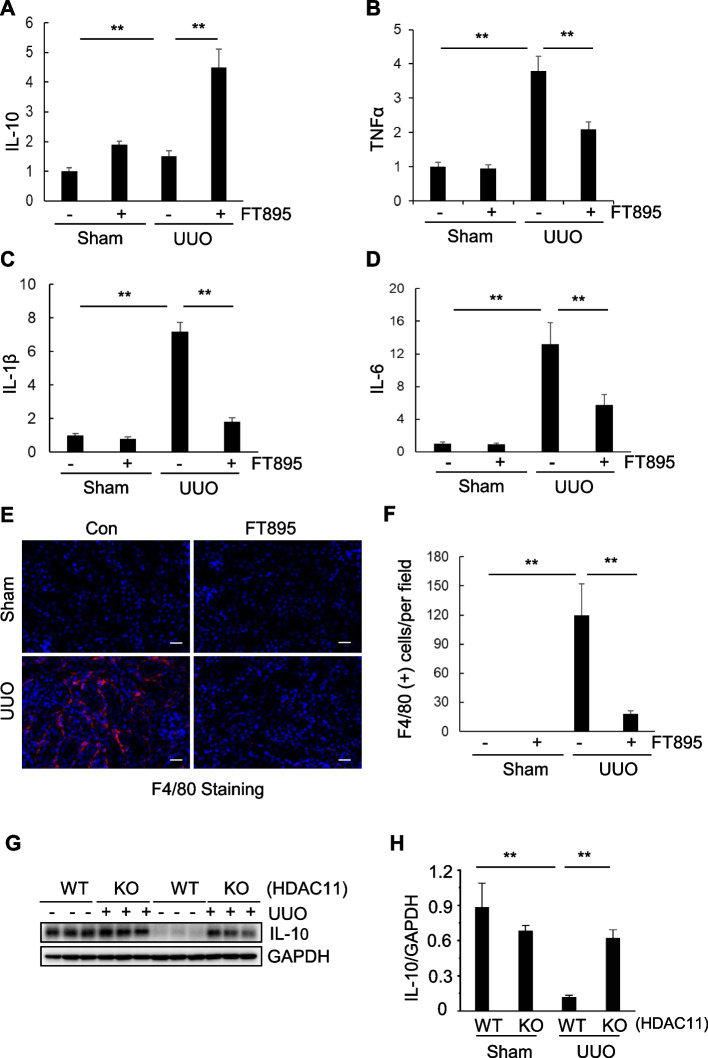
Inhibition of HDAC11 limits inflammation and macrophage infiltration in the kidneys after UUO injury. Quantitative PCR (qPCR) in the renal cortex was used to examine the messenger RNA (mRNA) expression of IL-10 (**A**), TNF-α (**B**), IL-1β (**C**) and IL-6 (**D**) in obstructed kidneys and their sham control. Data are represented as means ± SD (*n* = 6). ** *P* < 0.01, compared with groups indicated. **E** Immunostaining of F4-80 showed that macrophage infiltration was up-regulated in obstructed kidneys and considerably restricted in FT895 treated kidneys (original magnification 200 X). **F** Histogram shows quantification of the F4-80 macrophage was significantly upregulated in UUO mice compared with control mic and significantly decreased in FT895 treated group. Positively stained cells were counted in 10 fields, and mean numbers per field. Data are represented as means ± SD (*n* = 3). Kidney tissue lysates were prepared from wild-type (WT) and HDAC11 knockout (KO) mice and subjected to immunoblot analysis with antibodies against IL-10 and GAPDH (**G**). The expression levels of IL-10 were quantified by densitometry and normalized to GAPDH (**H**). Values are the mean ± SD (*n* = 6). **P < 0.01. Scale bars = 50 mm

Inflammation plays a crucial role in renal fibrosis. (Yeh et al. 2024) and HDAC11 was initially identified as a negative regulator of the anti-inflammatory cytokine IL-10 (Lai et al. 2011; Yanginlar and Logie 2018). To investigate whether inhibition of HDAC11 with FT895 reduces inflammation, we conducted qRT-PCR to analyze mRNA expression of IL-10 and several pro-inflammatory cytokines, including interleukin-1β (IL-1β), interleukin 6 (IL-6), and tumor necrosis factor- α(TNF-α). We found that mRNA expression for IL-1β, IL-6, and TNF-α was significantly increased in kidneys after UUO injury compared with sham control, IL-10 showed no significant change. Treatment with FT895 inhibited the upregulation of IL-1β, IL-6, and TNF-α and increased IL-10 in obstructed kidneys (Fig. 7A-D). We also examined immune cell infiltration using immunohistochemistry analysis with F4/80, a macrophage marker. Infiltration of F4/80 positive macrophages increased in the interstitial areas of the obstructed kidneys whereas FT895 treatment decreased the number of infiltrative macrophages. The cell counting results also showed that FT895 abolished UUO-induced increase in infiltrative macrophages (Fig. 7E, F).

To verify the role of HDAC11 in regulating IL-10 expression, we further examined the effect of global HDAC11 deletion on IL-10 protein levels in the kidney after UUO injury using immunoblot analysis. As shown in Fig. 7G-H, compared to WT mice, IL-10 expression was dramatically reduced in the UUO-injured kidneys of WT mice but was largely preserved in the injured kidneys of HDAC11 knockout mice. These findings are consistent with the role of HDAC11 in regulating IL-10 mRNA expression and suggest that HDAC11 acts as a negative regulator of IL-10 in the kidney following UUO injury.

In summary, HDAC11 activation is essential for promoting macrophage infiltration, inducing the expression of proinflammatory factors, and suppressing IL-10 in the kidneys following UUO injury.

## Discussion

HDAC11 is the sole member of the class IV HDAC subfamily and the most recently identified HDAC protein (Gao et al. 2002). It is predominantly expressed in the brain, skeletal muscle, heart, testis, and kidney (Gao et al. 2002). While previous studies have linked HDAC11 to brain degeneration, chronic muscle metabolic disease, myocarditis, and various tumors,(Chen et al. 2022; Khatun et al. 2024; Liu et al. 2020, 2023) its role in kidney diseases has been less explored. In this study, we found that HDAC11 was expressed in renal epithelial cells but not in interstitial fibroblasts. Both pharmacological and genetic inhibition of HDAC11 attenuated the development of renal fibrosis and pEMT following UUO injury. Furthermore, HDAC11 inhibition reduced the arrest of renal epithelial cells at the G2/M phase and suppressed the activation of several signaling pathways associated with pEMT induction and proinflammatory factor production. These findings suggest that HDAC11 may contribute to renal fibrosis by facilitating the development of a profibrotic phenotype in renal tubular cells.

The process of pEMT involves renal tubular cells losing epithelial characteristics and acquiring a mesenchymal phenotype, marked by the re-expression of proteins typically found in embryonic kidneys (Grande et al. 2015; Lovisa et al. 2016; Sheng and Zhuang 2020). These cells remain attached to the basement membrane without transforming into fibroblasts (Grande et al. 2015; Lovisa et al. 2016; Sheng and Zhuang 2020). However, renal epithelial cells undergoing pEMT are often arrested at the G2/M phase of the cell cycle, leading to a profibrotic phenotype that produces significant amounts of profibrotic and proinflammatory factors (Grande et al. 2015; Lovisa et al. 2016; Sheng and Zhuang 2020). These factors promote fibroblast conversion into myofibroblasts and trigger proinflammatory responses in the tubulointerstitium (Sheng and Zhuang 2020). Supporting this concept, our data indicated that inhibiting HDAC11 with FT895 or genetically deleting HDAC11 reduced fibronectin and collagen I expression while restoring E-cadherin levels in kidneys after fibrotic injury. Additionally, these treatments decreased the number of renal tubular cells expressing acetyl-histone H3pser10. Furthermore, HDAC11 inhibition suppressed UUO-induced upregulation of α-SMA, a marker for myofibroblasts (Humphreys et al. 2010; Sheng and Zhuang 2020). Since HDAC11 is not expressed in renal fibroblasts and complete EMT with α-SMA expression does not occur based on gene tracing observations (Humphreys et al. 2010), our findings suggest that HDAC11 inhibition reduces myofibroblast activation due to its regulatory role on pEMT during kidney injury.

HDAC11 may contribute to pEMT by upregulating the transcription factors Snail and Twist. Previous studies have shown that E-cadherin is a fibrosis suppressor protein, and the loss of its expression in association with the epithelial mesenchymal transition occurs during renal fibrosis (Grande et al. 2015; Lovisa et al. 2016; Sheng and Zhuang 2020). Since Snail and Twist are two transcription factors that mediates pEMT via suppression of E-cadherin expression, (Kalluri and Weinberg 2009). HDAC11 may recruit a repressor complex to the E-cadherin promoter, where it deacetylates histone H3 and H4 to create a repressive chromatin environment for Snail and Twist to exert their functional roles. In support of this hypothesis, it has been reported that treatment with a pan-histone deacetylase inhibitor Trichostatin A effectively abolishes the repressive effect of Snail, leading to increased dimethylation of histone H3 lysine 9 (H3K9me2), a mark associated with transcriptional repression at the E-cadherin promoter region in epithelial cells (Peinado et al. 2004). Here, we also observed that inhibition of HDAC11 with FT895 was accompanied by increased expression of H3K9me2 in RPTCs undergoing TGFβ1-induced EMT, suggesting a similar mechanism may be at play. Further investigation is required to determine whether the repressor complexes containing HDAC11 activity are involved in the transcriptional processes related to EMT.

Smad3 and STAT3 may also play a role in mediating HDAC11-induced EMT of renal epithelial cells. Previous studies have shown that activation of these two intracellular signaling pathways promotes renal tubular EMT and renal fibrosis (Pang et al. 2010; Wu et al. 2022). In the present study, we observed that inhibition of HDAC11 reduced the phosphorylation (activation) of both Smad3 and STAT3 in the kidney with UUO injury as well as in cultured renal epithelial cells stimulated with TGFβ1, highlighting the importance of HDCA11 in regulating their phosphorylation. However, the precise mechanism by which HDAC11-mediated deacetylation influences the phosphorylation of these signaling molecules remains unclear. Since Smad7 is an established inhibitor of Smad3, it is possible that HDAC11 may promote Smad3 activation by suppressing Smad7 expression. In this regard, it has been reported that HDAC5 can inhibit the transcriptional activity of myocyte enhancer factor 2 A, thereby repressing Smad7 transcription and leading to Smad3 activation (Xiang et al. 2024). Our recent studies also demonstrated that blocking class IIa HDACs with MC1568 increased Smad7 expression and decreased Smad3 expression in a murine model of UUO-induced renal fibrosis (Xiong et al. 2019). Consistent with these findings, we found that HDAC11 inhibition prevented UUO-induced downregulation of Smad7, suggesting that a similar mechanism may underlie inactivation of the Smad3 pathway in this context. Unlike Smad3, STAT3 can be directly acetylated by p300 and deacetylated by class I HDAC members, and its acetylation status can influence its phosphorylation levels (Ray et al. 2005; Zhuang 2013). For example, HDAC3-mediated deacetylation actyl-STAT3 is essential for STAT3(Y705) phosphorylation (Lu et al. 2018). Based on these insights, we speculate that HDAC11 may similarly act as a molecular switch in the STAT3 signaling cascade, linking HDAC11 activation to the development of EMT and renal fibrosis.

Finally, suppression of renoprotective molecules may constitute another mechanism by which HDAC11 promotes pEMT and renal fibrosis. In this context, some molecules, such as Klotho, BMP-7, and Krüppel-like factor 15 (KLF15), have been shown to protect against renal fibrosis in the kidney following chronic injury (Castillo 2023; Li et al. 2015; Wang et al. 2019) Among them, KLF15 is a transcription factor that suppresses renal fibrosis by inhibiting multiple signaling pathways such as the Wnt/β-catenin and TGF-β1/Smad3. (Gu et al. 2017; Lu et al. 2019). Recently, Mao et al. demonstrated that KLF15 is down-regulated in UUO kidneys, and HDAC11 knockdown or inhibition largely restores KLF15 expression in tubular epithelial cells or in the injured kidneys (Lu et al. 2019). This suggests that HDAC11 inhibition-mediated restoration of KLF15 may be required for protecting the kidney against the development of EMT and fibrosis. In addition to KLF15, other renoprotective molecules such as Klotho, BMP-7 are also subject to regulation by acetylation. (Shen and Zhuang 2022; Xiong et al. 2019), suggesting that HDAC11 may modulate their expression as well. Indeed, we found that the UUO-induced downregulation of these molecules was largely reversed by global deletion of HDAC11, indicating that HDAC11 may contribute to pEMT and renal fibrosis by suppressing the expression of multiple renoprotective factors.

Although the detailed mechanism by which HDAC11 contributes to G2/M phase cell cycle arrest of renal tubular cells remains unclear, emerging evidence suggests that HDAC11 acts as a regulator of the G2/M checkpoint in tumor cells by repressing mitotic drivers through epigenetic mechanisms, including deacetylation of key transcription factors and checkpoint proteins. For example, HDAC11 has been shown to promote hypoacetylation of E2F, a transcription factor that drives cell cycle progression in breast cancer cells (Feng et al. 2007). Acetylation of E2F generally enhances its transcriptional activity, promoting the expression of genes required for cell cycle progression. Conversely, its inactivation by hypoacetylation can lead to G2/M phase arrest through activation of the Cyclin B1–CDK1 complex, in a mechanism linked to STAT3 signaling (Fischer et al. 2022). Supporting this link, Sun et al. demonstrated that E2F is required for STAT3-mediated upregulation of Cyclin B1 and Cdc2, facilitating the G2/M transition (Sun et al. 2019). In line with these observations, we found that either pharmacological inhibition or genetic deletion of HDAC11 reduces STAT3 phoshorylation in the kidney following UUO injury, suggesting a similar mechanism may operate in renal tubular cells. It should be noted that G2/M arrest and pEMT are interconnected maladaptive responses of renal tubular cells to chronic injury — the arrested state stabilizes pEMT, while pEMT can induce cell cycle arrest; together they perpetuate the fibrotic microenvironment (Lovisa et al. 2015, 2016; Sheng and Zhuang 2020). In this context, HDAC11-mediated molecular events associated with pEMT, such as upregulation of Snail and Twist and activation of TGF-β signaling may also indirectly cause renal epithelial cells to arrest at the G2/M phase of the cell cycle in fibrotic injured kidney. Additional research is needed to explore these possibilities.

Moreover, HDAC11 may accelerate renal fibrosis by inducing renal inflammation. This is evident by our findings that (1) inhibition of HDAC11 blocked NF-κB phosphorylation, a post-translational modification that enhances transcriptional activity, and (2) reduced the expression of multiple proinflammatory cytokines (such as TNF-α, IL-1β, and IL-6) driven by NF-κB (Jimi et al. 2019) and infiltration of macrophages. To date, however, the mechanisms by which HDAC11 regulates NF-κB phosphorylation remain unknown. In other contexts, depletion of HDAC6 has been shown to up-regulate inhibitor of κB (IκB), thereby preventing nuclear translocation of NF-κB subunits and suppressing NF-κB reporter activation.(Barter et al. 2022). Furthermore, HDAC3 has been identified as a key regulator of NF-κB signaling, largely through deacetylation of the RelA/p65 subunit (Wang et al. 2024). Whether HDAC11 influences NF-κB activity by modulating HDAC3 expression or function, however, is currently unclear. At present, no direct evidence supports an HDAC11–HDAC3 regulatory axis in the kidney. Thus, although our data implicate HDAC11 in NF-κB–mediated inflammatory and fibrogenic responses, additional studies will be required to determine whether HDAC11 acts independently or in cooperation with HDAC3 to regulate NF-κB signaling during renal fibrosis.

In addition to its effect on NF-κB, HDAC11 may also mediate the expression of proinflammatory responses through suppression of IL-10. It has been reported that HDAC11 negatively regulated the expression of this cytokine in mouse and human in macrophages by interacting with the distal segment of the promoter region encoding IL-10 (Villagra et al. 2009). Since IL-10 is a cytokine with potent anti-inflammatory properties and administration of IL-10 suppresses chemokines, inflammation, and fibrosis in a model of chronic renal disease,(Mu et al. 2005) increased expression of IL-10 by HDAC11 inhibition may confer an anti-fibrotic effect via suppressing renal inflammation in the kidney following injury. Supporting this speculation, we observed that pharmacological inhibition of HDAC11 increased expression of IL-10 in UUO injured kidneys.

Emerging evidence has demonstrated the significant role of HDAC11 in regulating immune responses, metabolic processes, and tumorigenesis (Chen et al. 2022; Gao et al. 2002; Liu et al. 2020). Several potent and selective inhibitors of HDAC11 have been implicated in investigating the function of HDAC11 and its potential therapeutic applications.(Chen et al. 2022) FT895 is a novel and highly selective inhibitor (IC50 = 3 nM) for HDAC11 and is stable in serum (t1/2 = 9.4 h in mouse) (Martin et al. 2018). In addition to its effectiveness in renal fibrosis, FT895 has only been shown to induce thermogenesis to circumvent adipocyte catecholamine resistance. (Robinson et al. 2023) and potentiates the tumoricidal effects of cordycepin against malignant peripheral nerve sheath tumor in mice (Huang et al. 2022). Further investigation of FT-895’s role in various kidney diseases would provide more evidence for its potential use in the field of nephrology.

## Conclusions

**Fig. 8:**
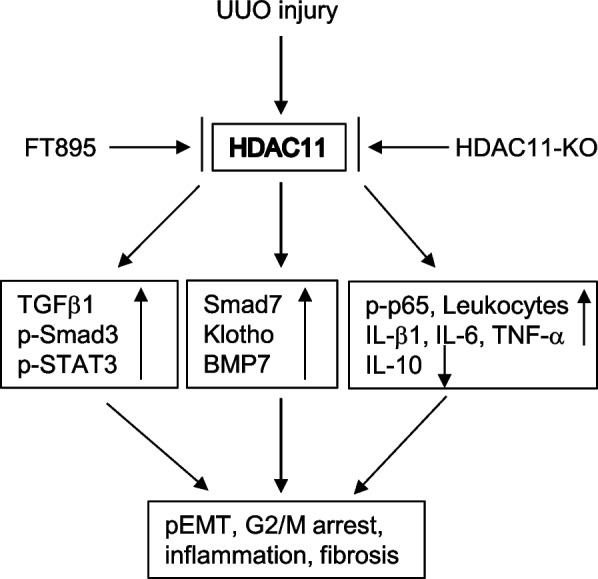
Schematic diagram for the role of HDAC11 in UUO-induced renal fibrosis. UUO injury to the kidney results in increased expression of HDAC11 in renal tubular cells. Elevated HDAC11 activity activates the TGFβ/Smad3 and STAT3 signaling pathways, downregulates Smad7, Klotho, and BMP-7, and induces NF-κB signaling. These changes promote the conversion of renal epithelial cells to a partial epithelial–mesenchymal transition (pEMT) phenotype, trigger G2/M phase cell cycle arrest, and enhance inflammation. Pharmacological or genetic inhibition of HDAC11 reverses these profibrotic responses and attenuates renal fibrosis

This study is the first to demonstrate the critical role of HDAC11 in promoting the dedifferentiation of renal epithelial cells into a profibrotic phenotype and driving the progression of renal fibrosis. These processes may occur through upregulation of Twist and Snail, activation of the Smad3, STAT3, and NF-κB signaling pathways, and suppression of multiple anti-fibrotic molecules such as Smad7, BMP-7, and Klotho (Fig. 8). Therefore, targeting HDAC11 may offer significant therapeutic potential for the treatment of chronic fibrotic kidney disease.

## Supplementary Information

Supplementary Material 1.

## Data Availability

No datasets were generated or analysed during the current study.

## Abbreviations

α‐SMA: α‐Smooth muscle actin
BMP7: Bone Morphogenetic Protein 7
CKD: Chronic kidney disease.
ECM: Extracellular matrix
FBS: Fetal bovine serum
GAPDH: Glyceraldehyde 3‐phosphate dehydrogenase
HDAC11: Histone deacetylase 11
H3Ser10: Phospho‐histone H3 at Ser10
KLF15: Kruppel-like factor 15
pEMT: Partial epithelial‐mesenchymal transition
RPTCs: Renal proximal tubular cells
siRNA: Small interfering RNA
STAT3: Signal transducer and activator of transcription
TGF-β: Transforming growth factor-β
