# Supplementary material for: HDAC11 promotes renal fibrosis by induing partial epithelial-mesenchymal transition and G2/M phase arrest in renal epithelial cells
Source: Mol Med. 2025 Nov 22;31:344. doi: 10.1186/s10020-025-01367-3 (PMC12755028; doi:10.1186/s10020-025-01367-3)
Supplement: Supplementary file 1 — Supplementary Material 1. [file 10020_2025_1367_MOESM1_ESM.pdf]

Supplementary Table 1. Details of primary antibodies used for immunoblotting analysis

Antibody	Catalogue number	Supplier	Dilution
α-SMA	ab5694	Abcam (Cambridge, MA, USA)	1:1000
Twist	ab175430	Abcam (Cambridge, MA, USA)	1:1000
Collagen-I	ab138492	Abcam (Cambridge, MA, USA)	1:1000
Snail	orb221325	Biorbyt (Durham, NC, USA)	1:1000
E-cadherin	14472	Cell Signaling Technology (Danvers, MA, USA)	1:1000
Acetyl-histone H3	9649s	Cell Signaling Technology (Danvers, MA, USA)	1:1000
p-Smad3	9520s	Cell Signaling Technology (Danvers, MA, USA)	1:1000
Smad3	9523s	Cell Signaling Technology (Danvers, MA, USA)	1:1000
p-Stat3	9145s	Cell Signaling Technology (Danvers, MA, USA)	1:1000
Stat3	12640s	Cell Signaling Technology (Danvers, MA, USA)	1:1000
p-NF-κBP65	3033s	Cell Signaling Technology (Danvers, MA, USA)	1:1000
NF-κBP65	8242s	Cell Signaling Technology (Danvers, MA, USA)	1:1000
Fibronectin	NBP1-91258	Novus Biologicals (Centennial, CO, USA)	1:1000
p-histone H3	NB21-1091	Novus Biologicals (Centennial, CO, USA)	1:1000
GAPDH	sc-137179	Santa Cruz Biotechnology (Dallas, TX, USA)	1:1000
α-Tubulin	sc-5286	Santa Cruz Biotechnology (Dallas, TX, USA)	1:1000
HDAC11	sc-390737	Santa Cruz Biotechnology (Dallas, TX, USA)	1:1000
Smad7	sc-365846	Santa Cruz Biotechnology (Dallas, TX, USA)	1:1000
Klotho	sc-515942	Santa Cruz Biotechnology (Dallas, TX, USA)	1:1000
BMP7	sc-365846	Santa Cruz Biotechnology (Dallas, TX, USA)	1:1000
Histone H3	07-108	Sigma-Aldrich (St. Louis, MO, USA)	1:1000

Supplemental Table 2. Details of primary antibodies used for immunofluorescence staining.

Antibody	Catalogue number	Supplier	Dilution
Acetyl–histone H3	9677	Cell Signaling Technology (Danvers, MA, USA)	1:200
HDAC11	sc-390737	Santa Cruz Biotechnology (Dallas, TX, USA)	1:100
F4/80	ab6640	Abcam (Cambridge, MA, USA)	1:200
α-SMA	ab5694	Abcam (Cambridge, MA, USA)	1:100
Collagen-I	ab138492	Abcam (Cambridge, MA, USA)	1:100
Fibronectin	NBP1-91258	Novus Biologicals (Centennial, CO, USA)	1:200

Supplemental Figure 1.

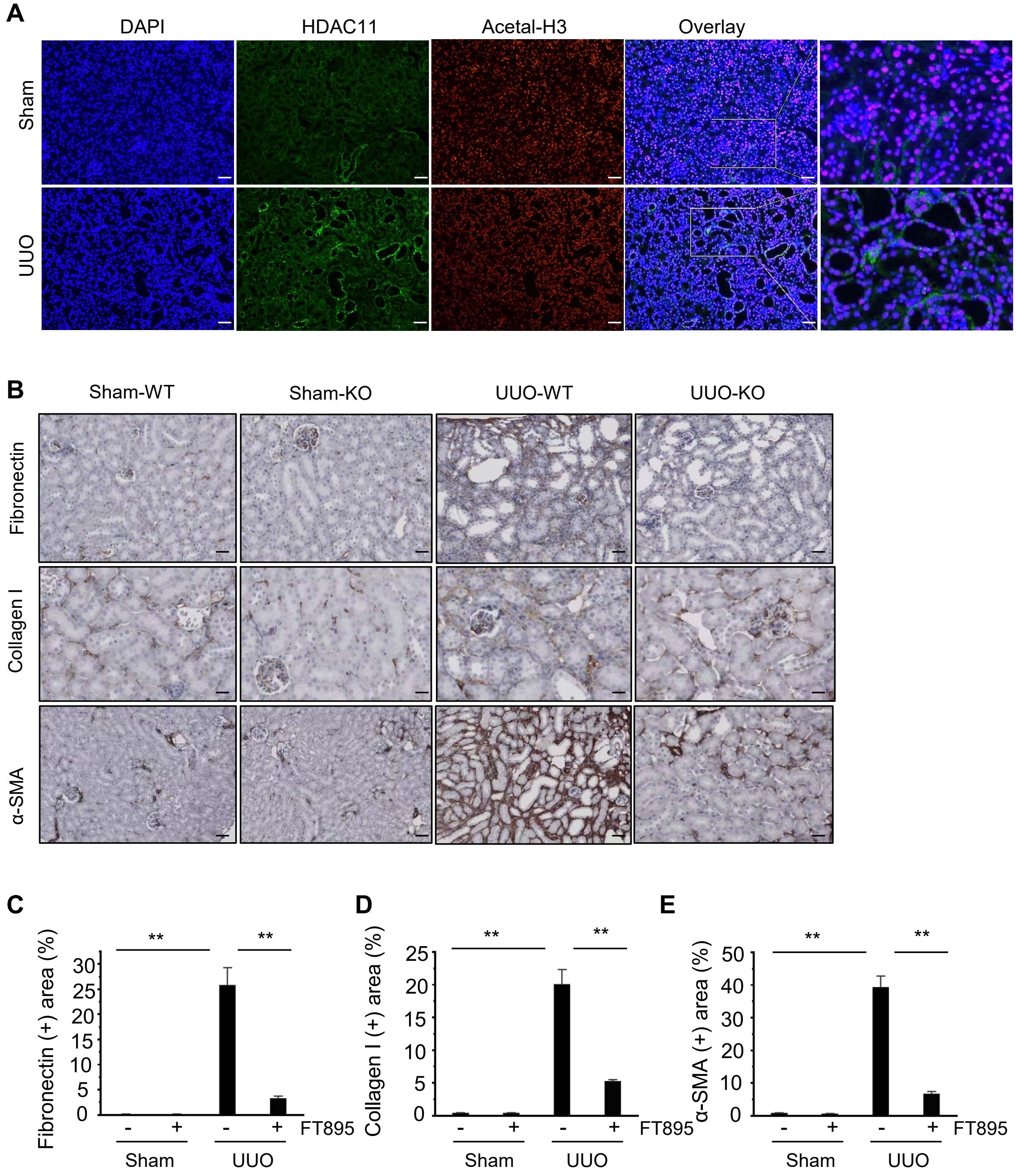

Supplemental Figure 1. Immunofluorescent staining of HDAC11 and acetyl-histone H3, with DAPI nuclear counterstaining, and immunohistochemical staining of fibronectin, collagen I, and α-SMA in kidneys following unilateral ureteral obstruction (UUO) injury. (A) Representative images show that global deletion of HDAC11 reduces UUO-induced HDAC11 expression and preserves acetyl-histone H3 levels in renal tubular cells. (B)

Immunohistochemical staining for fibronectin, collagen I, and α-SMA demonstrates increased deposition of extracellular matrix proteins and myofibroblast activation in UUO-injured kidneys compared with sham-operated controls (original magnification, 200 x). These changes are attenuated by global deletion of HDAC11, indicating that HDAC11 contributes to the development of renal fibrosis. Scale bar = 50 μm.

Supplemental Figure 2.

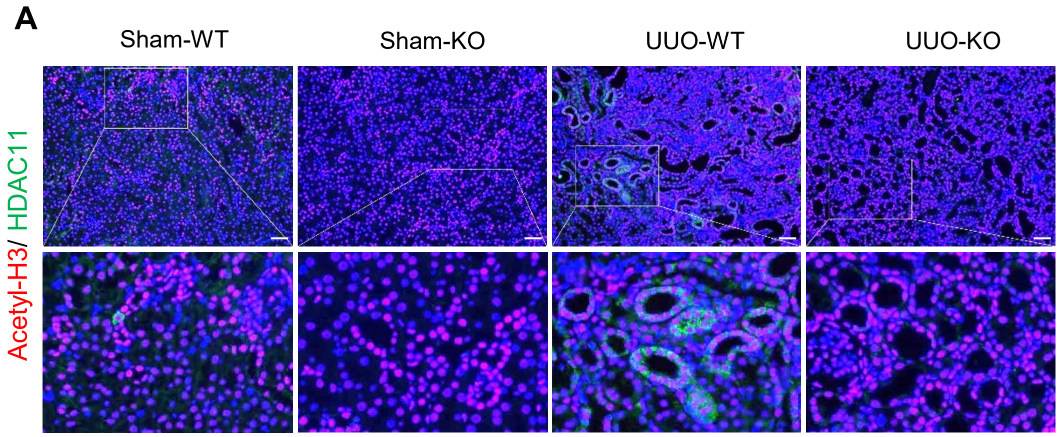

Supplemental Figure 2. Immunofluorescent staining of HDAC11 and acetyl-histone H3, with DAPI nuclear counterstaining, and immunohistochemical staining of fibronectin, collagen I, and α-SMA in kidneys following unilateral ureteral obstruction (UUO) injury. (A) Representative images show HDAC11 expression and acetyl-histone H3 levels in renal tubular cells after UUO(original magnification, 200 x). Scale bar = 50 μm.

Supplemental Figure 3.

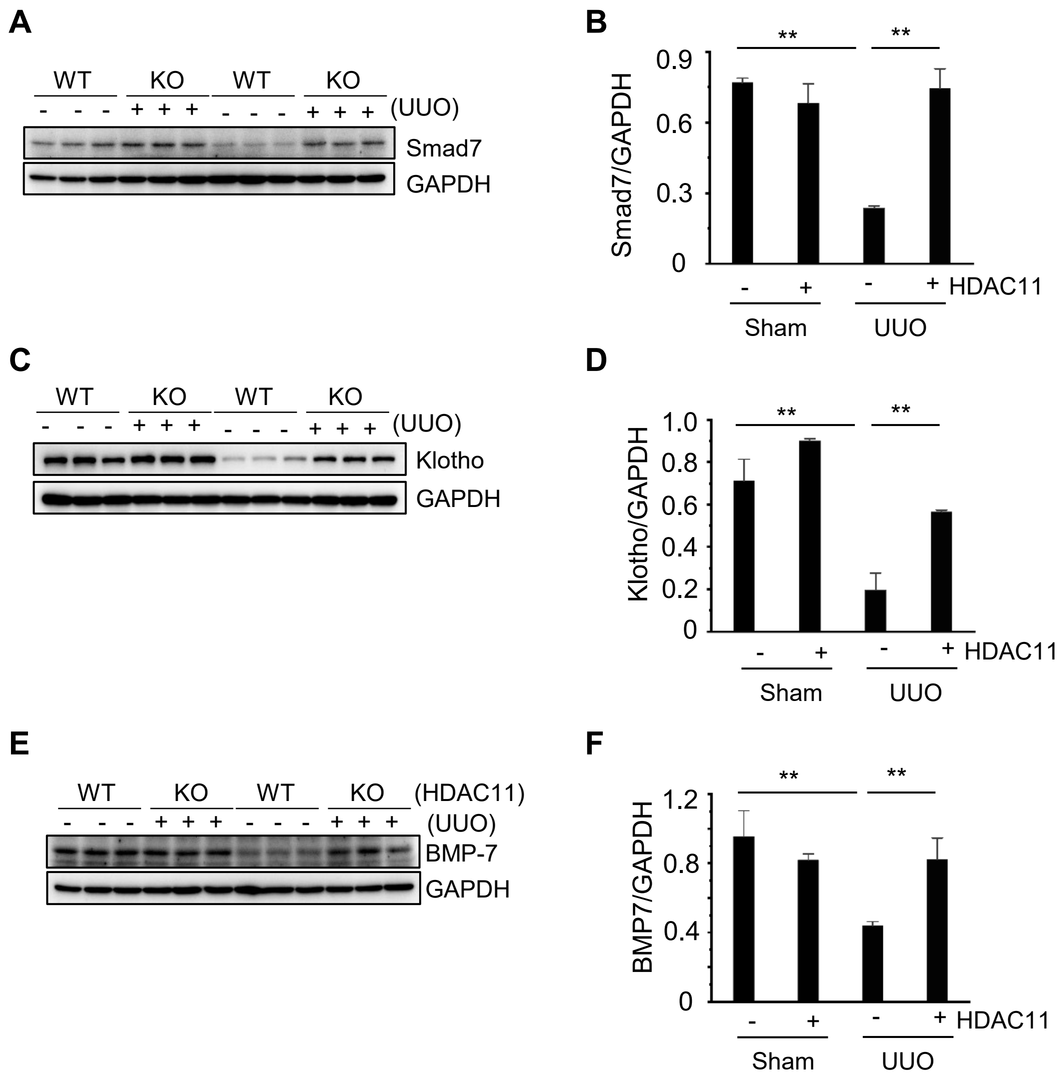

Supplemental Figure 3. Global deletion of HDAC11 restores the expression of Smad7, Klotho, and BMP-7 in the kidney following unilateral ureteral obstruction (UUO) injury. Kidney tissue lysates were prepared and subjected to immunoblot analysis with antibodies against Smad7 (A), Klotho (C), and BMP-7 (E). Protein levels were quantified by densitometry, and Smad7 (B), Klotho (D), and BMP-7 (F) were normalized to GAPDH. Values are presented as means ± SD (n = 6). *$P < 0.01$ compared with the indicated group.
